# A literature review of whether communication skills specific to psychiatry are being taught to medical undergraduates around the world

**DOI:** 10.1192/bjo.2021.797

**Published:** 2021-06-18

**Authors:** Sarah Winfield, Declan Hyland

**Affiliations:** 1 Locum SHO, Windsor House, Liverpool, Mersey Care NHS Foundation Trust; 2 Consultant Psychiatrist, Clock View Hospital, Liverpool, Mersey Care NHS Foundation Trust

## Abstract

**Aims:**

The ability to communicate effectively is an imperative skill for clinicians to master as doctor-patient communication is one of the most essential dynamics in health care. Patients with a mental disorder present a unique challenge for doctors with regards to effective communication due to the nature of their illness.

This literature review aimed to determine whether medical undergraduates around the world are taught psychiatric communication skills.

**Method:**

In January 2021, the following electronic databases were searched for articles relating to medical undergraduates, the concept of psychiatric communication skills and the teaching and support of such skill development: ERIC, MEDLINE, PsycINFO, SAGE and Web of Science. Combinations of keywords focussed the content of papers and truncation obtained alternative word endings. Generated articles were appraised iteratively for suitability against pre-defined inclusion criteria. The bibliographies of eligible articles were then examined to capture any further relevant studies. Ethical approval was not required.

**Result:**

1040 citations of potential relevance were initially identified. Following an iterative screening process, 10 articles (from seven different countries) were eligible for inclusion. 70% of papers used the modality of simulated patients to teach psychiatric communication skills and Technology Enhanced Learning (TEL) was used to create “virtual patients” for undergraduates to engage with. Discussing sensitive and emotive topics, such as suicide attempts or substance misuse, was less commonly taught compared to conditions such as anxiety and depression. Only 10% of papers explicitly taught medical undergraduates empathy or written communication skills and the importance placed on psychiatric teaching differed between countries.

**Conclusion:**

This literature review showed that some medical undergraduates receive psychiatric communication skills teaching, but the format and content of this varies. Increased consideration of incorporating TEL into psychiatric communications skills teaching is pertinent given undergraduates’ reduced face-to-face patient contact during the COVID-19 pandemic, but further work is needed to validate such technology. Written communication skills are rarely taught but are imperative given the high volume of written correspondence in clinical practice. Delivering such teaching is feasible and should be incorporated into undergraduate curricula. Medical educators need to consider cultural differences when developing psychiatric communication skills teaching. Cultural influences not only affect undergraduate perceptions of psychiatry and mental illness, but also a patient's understanding and interpretation of their illness experience. Medical undergraduates may come from various cultural backgrounds, so actively discussing these differences opportunistically may augment the ability of medical undergraduates to be empathetic and establish therapeutic rapport with patients with mental illness.

